# The efficacy and safety of acupoint catgut embedding therapy for depression: a protocol for systematic review and meta-analysis

**DOI:** 10.3389/fpsyt.2023.1331780

**Published:** 2024-01-08

**Authors:** Yadi Li, Jianlong Zhou, Zheng Wei, Xia He, Lizhu Liang, Kejimu Sunzi

**Affiliations:** People’s Hospital of Deyang City, Deyang, Sichuan, China

**Keywords:** acupoint catgut embedding, acupuncture, depression, study protocol, meta-analysis, systematic review

## Abstract

**Background:**

Depression is a common public health problem, characterized by persistent low mood, lack of pleasure and exhaustion. Conventional treatments such as antidepressants and psychotherapy have some limitations, including variable efficacy, adverse side effects and high costs. Acupoint catgut embedding (ACE) therapy, as a subtype of acupuncture, has gained increasing clinical application due to its long-term effects, higher patient compliance, and cost-effectiveness. This study aims to conduct a meta-analysis to evaluate the efficacy and safety of ACE for depression.

**Methods:**

Electronic searches will be conducted in 12 databases (both in English and Chinese databases), encompassing from inception to April 2022, without language restrictions. Randomized controlled trials (RCTs) that involve ACE for treating depression will be included. The primary outcome measures will include the response rate, Hamilton Depression Rating Scale (HAMD), Beck Depression Inventory (BDI), and Traditional Chinese Medicine Symptom Scale (TCMSSS). The secondary outcome measure will include Quality of Life scale score (QoL) and the incidence of adverse events. Results will be presented as risk ratios for dichotomous data and mean differences for continuous data. Two reviewers will independently conduct study selection, data extraction, and quality assessment. The methodological quality of eligible studies will be evaluated according to the criteria specified by the Cochrane Handbook for Systematic Reviews of Interventions (Version 5.1.0). Meta-analysis will be performed by RevMan 5.3 software.

**Discussion:**

Due to the limitations, a safer, high-efficacy and non-pharmacological intervention with minimal side effects is required for treating depression. ACE has the advantages of longer-lasting effects, improved patient compliance, and reduced treatment costs. This protocol represents a meta-analysis and systematic review, aiming to present the current evidence regarding the efficacy and safety of ACE for depression. It seeks to provide clinicians with a theoretical basis and valuable references for complementary and alternative medicine therapies in their treatment approaches.

**Systematic review registration:**

https://www.crd.york.ac.uk/PROSPERO/#recordDetails, Identifier CRD42022325966.

## 1 Introduction

Depression is a prevalent mental disorder characterized by persistent low mood, lack of pleasure, and exhaustion (1). As a major public health problem, it exerts a substantial impact on individuals and society (2), affecting a significant portion of the global population. According to the World Health Organization, more than 350 million individuals suffer from depression worldwide, with this number increasing annually (3, 4). Global estimates indicate that 3.2% of individuals are currently depressed, and the rate has risen by 27.6% due to the COVID-19 pandemic (5). In the United States, the lifetime prevalence of major depressive disorder (MDD) has been reported at 20.6% (6). MDD was identified as a significant contributor to the burden of suicide and ischemic heart disease in the 2010 Global Burden of Disease study (7). Beyond the personal distress experienced by individuals with severe depression, there is a considerable socioeconomic burden associated with this mental illness, resulting in collective costs ranging from US$29 to US$48 billion in the United States alone (8). Such costs are attributed to increased unemployment, disability, and reduced work performance (9). Disturbingly, over 10% of depressed individuals experience suicidal thoughts, with some expressing intention to act (10), leading to a lifetime suicide rate ranging from 3.8 to 7.8% (11). More alarmingly, studies have projected that by 2030, depression will become the most common disabling condition in the world, ranking as the second leading cause of disability (2, 12, 13).

Currently, the recommended treatments for depression include antidepressants, psychological interventions, and adjunctive or combined pharmacological and/or psychological treatments (12, 14). However, despite the potential benefits of these treatments, more than one-third of patients do not respond adequately or only achieve partial response (4, 14, 15), indicating the limitations of standard drug interventions (16). Additionally, unstable efficacy and intolerable side effects of antidepressant medication are commonly reported (17, 18). While psychotherapy has shown equivalent outcomes to antidepressant medication, its acceptance remains uneven (19), and the dropout rates for both treatments are similar (20). Furthermore, the high costs and adverse side effects further limit the clinical efficacy of these conventional therapies (21). In light of these limitations, there is an urgent need to develop safer, high-efficacy, non-pharmacological interventions with minimal side effects for treating depression.

Complementary and alternative medicine (CAM) therapies are frequently used in the treatment of depression (22–24), either as adjuncts or replacements for conventional approaches (22). Among these CAM therapies, acupuncture is commonly utilized in China (25), and its popularity is growing in the United States, the United Kingdom, Japan, and Korea (26–28). Current research suggests that acupuncture may have therapeutic benefits in addition to SSRIs alone for patients with moderate to severe depression and is generally well-tolerated (29). The overall efficacy of various acupuncture methods after 6 weeks was approximately 70 to 83.7%, which was equivalent to or even higher than antidepressant drugs (30). Similarly, several high-quality randomized controlled trials (RCTs) also found that acupuncture may be not only a safe adjunctive treatment with antidepressants but also more effective in improving depressive symptoms (31–33). A meta-analysis revealed that acupuncture can significantly reduce the severity of depression, with a strong correlation between increased acupuncture sessions and decreased depression severity (2). With its advantages of availability, affordability, and rare serious adverse events, acupuncture has gained widespread acceptance among patients and is extensively used worldwide (28, 34–36).

In traditional Chinese Medicine, it holds the theory that a state of health is maintained by a balance of energy within the body (37). Acupuncture may correct the imbalance of energy within the body by insertion of fine needles into different acupoints of the body. Recent studies have shown that acupuncture may treat depression by affecting various neurotransmitters, predominantly the endogenous opioid mechanism (EOM), catecholamines, serotonin and approximately 20 to 30 other neuropeptides (38). It has been found to regulate mitochondrial homeostasis, reduce depression-like behavior, regulate signal pathways and key proteins (39). Additionally, it can enhance brain regions and network neuroplasticity, decrease brain inflammation, potentially alleviate depressive disorder (40). Furthermore, similar to antidepressant medications, acupuncture can influence the neurotransmitter levels of serotonin and noradrenaline, along with the adenylate cyclase cyclic adenosine monophosphate-protein kinase A (AC-cAMP-PKA) cascade within the central nervous system (38).

Acupoint catgut embedding (ACE), as a subtype of acupuncture, has increasingly been used in clinical treatment in China in recent years. During ACE therapy, bioprotein catgut is embedded into acupoints using a disposable sterile needle, providing continuous and sustained stimulation to the acupoints for a week or longer (41). This therapy offers the meaningful benefit of reducing the frequency of patients seeking medical treatment, thereby conserving medical resources to some extent. Studies have indicated that ACE exhibits significant effects on chronic diseases (42, 43), and it has the advantages of easy operation and durable stimulation (44). Furthermore, the treatment costs of ACE have been reported to be lower than those of electroacupuncture (45).

Despite the growing number of studies focusing on ACE therapy for depression, there is currently limited literature available in English databases (46). Moreover, the clinical efficacy and safety of this treatment have been inconsistently reported, and there is a lack of large-sample, multicenter, RCTs providing high-quality clinical research evidence. Therefore, there is an urgent need to identify, evaluate, grade, and summarize the existing clinical evidence to provide a solid theoretical basis for clinical applications.

Consequently, this meta-analysis and systematic review will be conducted to evaluate and summarize the efficacy and safety of ACE therapy for depression. As the first meta-analysis of ACE therapy for depression, this review aims to explore the clinical effects and adverse reactions based on the analysis of suitable treatment paradigms in RCTs.

## 2 Methods and analysis

The methodology of this study will be conducted following the Preferred Reporting Items for Systematic Reviews and Meta-Analyses (PRISMA) extension statements. This protocol has been prepared in accordance with the recommendations of Cochrane, registered in PROSPERO (CRD42022325966).

### 2.1 Objectives

The aims of this meta-analysis and systematic review are described as following:

1. To evaluate the effectiveness and safety of acupoint catgut embedding (ACE) for depression in comparison to comfort therapy (such as placebo, sham catgut embedding, or blank control) or other therapies (such as Western medicine, traditional Chinese medicine, or non-drug therapy, etc.). The findings will serve to inform and facilitate clinical decision-making in practice.
2. To assess the methodological quality and the strength of evidence regarding ACE for depression, providing a comprehensive evaluation of the available research.
3. To determine the appropriate modality and frequency of intervention, and if possible, identify the optimal course of treatment for depression using ACE therapy.

### 2.2 Eligibility criteria

#### 2.2.1 Types of studies

(1) All RCTs focusing on the treatment of depression using ACE therapy.
(2) Studies involving single ACE therapy, as well as those comparing ACE with other interventions.
(3) Studies published in English or Chinese due to limited financial resources for translation.
(4) Studies published between the database’s establishment and April 2022.
(5) There will be no restrictions on language or publication type.

#### 2.2.2 Types of participants

(1) Participants diagnosed with depression who have been enrolled in the RCTs.
(2) There will be no restrictions on gender, age, race, onset time, or source of cases.

#### 2.2.3 Types of intervention

(1) The intervention in the treatment group will include:

- Simple acupoint catgut embedding.

(2) The interventions in the control group will include:

- Comfort therapy (placebo, sham catgut embedding, or blank control).
- Other therapies (Western medicine, traditional Chinese medicine, other types of acupuncture, or non-drug therapy).

(3) The specific materials used for acupoint catgut embedding will not be considered in this review.

#### 2.2.4 Types of outcome measures

(1) The primary outcomes will include the following measures:

- Response rate In this study, the response rate is defined as achieving a specific percentage reduction in symptoms:

1) A reduction greater than 50%, or;
2) The complete elimination of symptoms, i.e., meeting the criteria for complete remission.

- Hamilton Depression Rating Scale score (HAMD), Beck Depression Inventory (BDI), etc.
- Traditional Chinese Medicine Syndrome Score Scale (TCMSSS)

The secondary outcome will focus on safety and include the following:

- Quality of Life scale score (QoL)
- Reported incidence of all adverse events, such as sharp pain, subcutaneous hematoma, fatigue, palpitations, fever, infection, and other relevant adverse reactions.

#### 2.2.5 Exclusion criteria

(1) Non-RCTs, such as reviews, commentary articles, letters, case reports, etc.
(2) Duplicate publications.
(3) Studies that do not explicitly state the use of ACE therapy or that combine it with other treatments.
(4) Studies found in grey literature.
(5) Studies with incomplete data.

### 2.3 Electronic searches

A search strategy will be designed and conducted following the guidelines outlined in the Cochrane Handbook (47). The meta-analysis will be reported based on the PRISMA (Preferred Reporting Items for Systematic Reviews and Meta-analyses) guidelines.

Electronic searches will be carried out by two researchers (YDL & KJMSZ) from the project’s initiation until April 2022. English databases (PubMed, Cochrane Library, Web of Science, Embase, OVID, Scopus) and Chinese databases (China National Knowledge Infrastructure (CNKI), Wan-Fang Data (WANFANG), Chinese Biomedical Literature Database (CBM), Chinese Scientific Journal Database (VIP), and Duxiu Database) will be included in the search. The Chinese Clinical Trial Registry Center will also be searched for ongoing trials. There will be no restrictions on countries or publication types.

The search will utilize keywords such as acupoint catgut embedding, catgut implantation, catgut embedding, depression, depressive symptoms, depressive symptom, and emotional depression. A combination of subject and free terms will be employed to ensure a comprehensive search, irrespective of language or publication type. The search will be conducted across all databases to ensure the identification of all relevant articles. An example search strategy for the PubMed database is provided in Table 1.

**Table 1:** Search strategy in PubMed database.

Search step	Search query
#1	“Depression”[MeSH Terms]
#2	“depressive symptoms”[Title/Abstract] OR “depressive symptom”[Title/Abstract] OR “symptom depressive”[Title/Abstract] OR “symptoms depressive”[Title/Abstract] OR “emotional depression”[Title/Abstract] OR “depression emotional”[Title/Abstract]
#3	#1 OR #2
#4	“acupoint catgut embedding”[Title/Abstract] OR “catgut implantation”[Title/Abstract] OR “catgut embedding”[Title/Abstract]
#5	“randomized controlled trial”[Publication Type] OR “randomized”[Title/Abstract] OR “placebo”[Title/Abstract]
#6	#3 AND #4 AND #5

Similar search strategies will be employed for the other databases. Additionally, in the case of studies without the full text, efforts will be made to contact the first author or corresponding author to obtain the necessary full text.

### 2.4 Data collection

**Figure 1:**
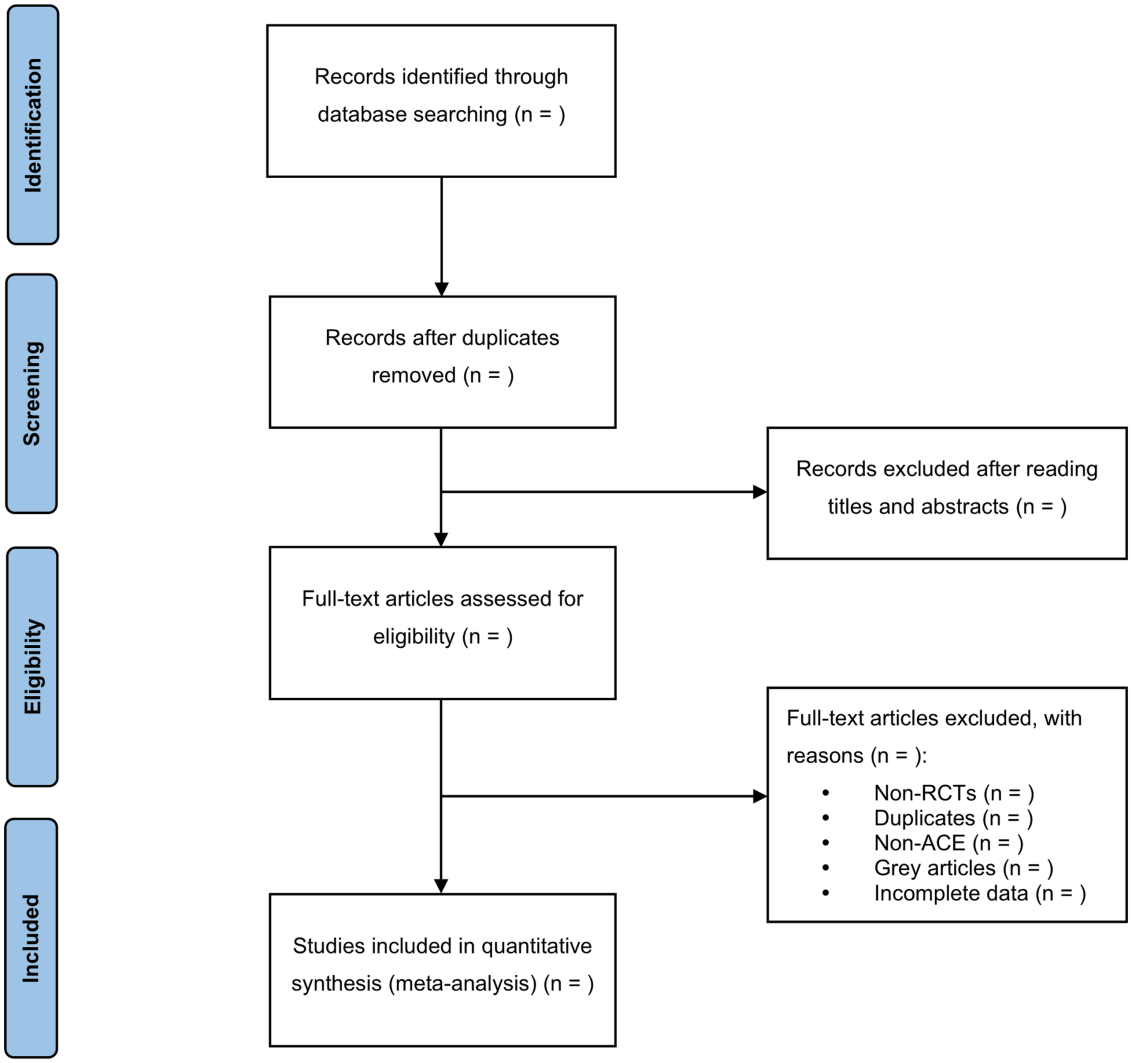
PRISMA flow chart.

In this step, the EndNote X9 software will be utilized to screen the search results. Two reviewers (YDL & KJMSZ) will independently conduct the screening process and cross-check the results. Based on the predefined inclusion and exclusion criteria, relevant studies will be selected after reviewing their titles, abstracts, or full texts. In the case of duplicate publications, the original publication will be chosen. If there are any incomplete data or ambiguous details, we will make efforts to contact the first author or corresponding author for clarification. In situations where disagreements arise, a third reviewer (JLZ) will be consulted, and the final decision will be made after discussion. A comprehensive overview of the selection process will be depicted in the PRISMA flow chart (Figure 1).

### 2.5 Data extraction and coding

Two reviewers (YDL & KJMSZ) will independently complete the data extraction using the provided data extraction form (Appendix 1). Any discrepancies that arise during the process will be resolved through discussion until a consensus is reached, or if necessary, by seeking the opinion of a third reviewer.

The following information will be recorded using a standardized data extraction form:

(1) Basic information of the study: First author, year, and country of publication.
(2) Study characteristics: Study inclusion criteria, participants’ details (age, gender, complications, severity, and course of depression).
(3) Details of study intervention(s) and control intervention(s) (modality, frequency, and duration).
(4) Risk of bias assessment.
(5) Outcome data, adverse effects, and drop-out rate.

EndNote software will be employed to help the reviewers manage data and identify any duplicate publications. In cases where two or more reports describe a single trial, only one publication will be included in the analysis.

#### 2.5.1 Assessing risk of bias in included studies

The methodological quality of eligible studies will be assessed using the Cochrane Collaboration Risk of Bias Tool (48). Two reviewers (YDL & KJMSZ) will independently evaluate the risk of bias for each included RCT based on the Cochrane Handbook V.5.1.0. The assessment will cover the following aspects:

(1) Random sequence generation.
(2) Allocation concealment.
(3) Blinding of participants and personnel.
(4) Blinding of outcome assessment.
(5) Incomplete outcome data.
(6) Selective reporting.
(7) Other potential sources of bias.

Each study will be categorized as having a high, low, or unclear risk of bias based on the evaluation. In case of disagreements, a third reviewer (JLZ) will be involved to assist in reaching a consensus.

### 2.6 Measures of treatment effect

Statistical analysis will be conducted using RevMan 5.3, and Forest plots will be employed to visually represent the relative strength of the treatment effects. For continuous variables, the mean difference (MD) will be used to express the treatment effect, while for dichotomous data, the risk ratio (RR) will be used. The estimated value and 95% confidence intervals for each effect will be calculated and presented in the analysis.

### 2.7 Dealing with missing data

In the event of missing or ambiguous data in the included studies, efforts will be made to contact the first author or corresponding author for assistance. If the missing data cannot be obtained despite attempts to contact the authors, the study will be excluded from the analysis.

For studies with missing data, the intention-to-treat (ITT) analysis will be employed to conduct statistical and omission analysis. ITT analysis helps maintain the integrity of the original randomized allocation of participants, even in cases where some data may be missing. This approach ensures that participants are analyzed according to their original randomized groups, regardless of any missing data.

### 2.8 Assessment of heterogeneity

Heterogeneity among the included studies will be assessed. The Mantel–Haenszel χ^2^ test (with a significance level of *α* = 0.1) and the *I^2^* statistic will be used to determine the extent of heterogeneity (49). Based on the test results, either the fixed-effects model or the random-effects model will be chosen for the data calculation.

For cases of substantial clinical heterogeneity, subgroup analysis will be conducted to investigate the potential reasons behind the observed differences (50). This will aid in the interpretation of the data and provide further insights into the variability among the included studies.

### 2.9 Assessment of reporting biases

If there are 10 or more trials included in the subsequent meta-analysis, a funnel plot will be used to evaluate the potential publication bias. Funnel plots are commonly employed to visually assess the symmetry of data points, which can indicate whether there is a bias in the reporting of study results. Asymmetry in the funnel plot may suggest publication bias, where studies with statistically significant results are more likely to be published than those with non-significant results.

### 2.10 Data analysis

RevMan (Version 5.3) will be utilized to calculate the risk ratio (RR) for dichotomous data and the mean difference (MD) for continuous variables. The estimated value and 95% confidence interval (CI) of each effect will be calculated.

If the research results exhibit low heterogeneity (*I^2^* < 50%), a fixed-effect model will be used for the meta-analysis. Conversely, if the research results display significant heterogeneity (*I^2^* > 50%), a meta-analysis will be conducted using a random-effects model, following further analysis to understand the sources of heterogeneity.

In cases where the data are not suitable for quantitative synthesis, a narrative summary will be provided to present the findings of the included publications.

For trials reporting only pre- and post-intervention values, the mean changes will be calculated by subtracting the pre-measurements from the post-measurements. The standard deviation (s.d.) for changes will be estimated accordingly.

### 2.11 Subgroup analysis

In the presence of significant heterogeneity, a subgroup analysis will be conducted to explore the potential sources of heterogeneity. The following aspects will be considered as potential subgroup factors: age, gender, modality of intervention, and degree of depression.

If there is sufficient data available for each subgroup, a quantitative subgroup analysis will be performed to investigate the impact of these factors on the treatment effects.

However, if the data for subgroup analysis are insufficient, a qualitative synthesis will be conducted, where the findings from individual studies will be summarized and compared without a formal quantitative combination.

### 2.12 Sensitivity analysis

If significant heterogeneity persists even after conducting the subgroup analysis, a sensitivity analysis will be carried out. In the sensitivity analysis, the meta-analysis will be repeated after excluding low-quality studies or studies with potential sources of bias.

By performing the sensitivity analysis and comparing the results of the two meta-analyses, we can assess the robustness and reliability of the findings. This process helps to evaluate the impact of individual studies on the overall results and provides a more comprehensive understanding of the meta-analysis outcomes.

### 2.13 Summary of evidence

The researchers will assess the quality of evidence using the Grading of Recommendations, Assessment, Development, and Evaluation (GRADE) approach. The quality of evidence will be categorized into four levels: very low, low, moderate, or high.

Using the GRADE methodology allows for a systematic and transparent evaluation of the certainty and strength of the evidence, enabling the researchers to present a comprehensive summary of the evidence in a clear and reliable manner.

### 2.14 Patient and public involvement

Patients and/or the public were not involved in the design, conduct, reporting, or dissemination plans of this study.

### 2.15 Publishing the protocol

The findings of this systematic review will be disseminated through publication in a peer-reviewed journal, relevant conferences, and national and international meetings.

Publishing the protocol of this meta-analysis report prior to conducting the review offers several advantages. It enhances the transparency of the review methodology and allows for peer review feedback and comments before the actual review is initiated, thereby contributing to the improvement of the review’s overall quality and credibility. Additionally, publication of the protocol helps prevent duplication of efforts in case other researchers plan to conduct a similar review.

## 3 Discussion

Depression is a prevalent and burdensome disease, significantly impacting patients’ quality of life and imposing a substantial financial burden. Currently, conventional treatments such as antidepressants and psychotherapy are commonly used (12, 14, 17, 51). However, these treatments have drawbacks, including variable efficacy, adverse side effects, and high costs. As a result, there is growing interest in complementary and alternative therapies, with acupuncture emerging as a widely recognized approach for depression treatment worldwide in recent years. While acupuncture offers effectiveness, affordability, and a favorable safety profile, its frequent treatment sessions may impact patient compliance. In contrast, ACE as a subtype of acupuncture, offers the advantages of longer-lasting effects, improved patient compliance, and reduced treatment costs.

This protocol presents a systematic review that represents the first quantitative analysis aimed at exploring the efficacy and safety of ACE therapy for depression. By evaluating the evidence from published RCTs, we aim to provide a comprehensive understanding of the effects of ACE therapy in treating depression. Additionally, we seek to identify the appropriate modality, frequency of intervention, and treatment course for optimal outcomes.

The outcomes of this meta-analysis and systematic review are expected to offer valuable insights to clinicians, providing a solid theoretical basis and essential references for CAM therapies in depression treatment. Such evidence may significantly contribute to informed clinical decision-making when selecting treatment options for patients with depression.

If necessary, any amendments made to this protocol will be clearly documented, including the date of each amendment, a description of the changes, and the corresponding rationale.

### Protocol dissemination

We will disseminate the findings of this comprehensive study by publishing them in peer-reviewed journals. The publication of this comprehensive report will improve the transparency of the review methodology and obtain peer review feedback and comments before the review begins, which will also improve the quality and credibility of the review.

### Scope statement

Depression is a common mental disorder that has become a growing concern worldwide. Therefore, finding effective and safe treatments for depression is imperative. Acupoint catgut embedding therapy, as a subtype of acupuncture, has a long history in East Asia. It has gained increasing clinical application due to its long-term effects, higher patient compliance, and cost-effectiveness. However, its efficacy and safety for treating depression have not been comprehensively reviewed. This paper aims to fill this gap by providing a protocol for a systematic review and meta-analysis that will evaluate existing research in the field. Additionally, it will provide clinicians with a theoretical basis and valuable references for complementary and alternative medicine therapies for depression in their treatment approaches, inform and facilitate clinical decision-making in practice.

## Author contributions

YL: Data curation, Formal analysis, Methodology, Project administration, Resources, Supervision, Validation, Visualization, Writing – original draft, Writing – review & editing, Conceptualization, Software. JZ: Data curation, Formal analysis, Methodology, Software, Writing – original draft. ZW: Data curation, Software, Writing – original draft, Resources. XH: Data curation, Formal analysis, Methodology, Resources, Writing – original draft. LL: Supervision, Validation, Visualization, Writing – review & editing. KS: Supervision, Validation, Visualization, Writing – review & editing, Data curation, Project administration.

## Glossary

**Table:** 

ACE	acupoint catgut embedding
HAMD	Hamilton Depression Rating Scale
BDI	Beck Depression Inventory
TCMSSS	Traditional Chinese Medicine Symptom Scale
QoL	Quality of Life scale score
MDD	major depressive disorder
CAM	complementary and alternative medicine
RCTs	randomized controlled clinical trials
EOM	endogenous opioid mechanism
AC-cAMP-PKA	adenylate cyclase cyclic adenosine monophosphate-protein kinase A
PRISMA	Preferred Reporting Items for Systematic Reviews and Meta-Analyses
CNKI	China National Knowledge Infrastructure
WANFANG	Wan-Fang Data
CBM	Chinese Biomedical Literature Database
VIP	Chinese Scientific Journal Database
MD	mean difference
RR	risk ratio
ITT	intention-to-treat
CI	confidence interval
GRADE	Grading of Recommendations, Assessment, Development, and Evaluation
