# Supplementary material for: The efficacy and safety of acupoint catgut embedding therapy for depression: a protocol for systematic review and meta-analysis
Source: Front Psychiatry. 2024 Jan 8;14:1331780. doi: 10.3389/fpsyt.2023.1331780 (PMC10800672; doi:10.3389/fpsyt.2023.1331780)
Supplement: Supplementary file 1 [file Table_1.DOCX]

**The efficacy and safety of acupoint catgut embedding therapy for depression: a protocol for systematic review and meta-analysis**

**Appendix 1: Data Extraction Tool**

| Author and Date | Country | Sample size | Diagnosis for Inclusion | Severity of depression (mild/middle/severe) | Patients age | course of disease | Blinding | Intervention | Treatment frequency (treatment period) | Treated acupoints | Outcome measures | Adverse effects | Drop-out rate |
| --- | --- | --- | --- | --- | --- | --- | --- | --- | --- | --- | --- | --- | --- |
|  |  |  |  |  |  |  |  |  |  |  |  |  |  |
|  |  |  |  |  |  |  |  |  |  |  |  |  |  |
|  |  |  |  |  |  |  |  |  |  |  |  |  |  |
|  |  |  |  |  |  |  |  |  |  |  |  |  |  |
